# Supplementary material for: Motoneuron-driven computational muscle modelling with motor unit resolution and subject-specific musculoskeletal anatomy
Source: PLoS Comput Biol. 2023 Dec 7;19(12):e1011606. doi: 10.1371/journal.pcbi.1011606 (PMC10729998; doi:10.1371/journal.pcbi.1011606)
Supplement: S1 Text — (PDF) [file pcbi.1011606.s001.pdf]

SUPPORTING INFORMATION FILE

S1_Text

Motoneuron-driven computational muscle modelling with motor unit resolution and subject-specific musculoskeletal anatomy

Arnault H. Caillet[1,2,*], Andrew T.M. Phillips[1], Dario Farina[2,*], Luca Modenese[1,3,*]

1.Department of Civil and Environmental Engineering, Imperial College London, UK

2.Department of Bioengineering, Imperial College London, UK

3.Graduate School of Biomedical Engineering, University of New South Wales, Sydney, Australia

*d.farina@imperial.ac.uk (DF) l.modenese@unsw.edu.au (LM)

Contents

1. DERIVATION OF THE EXPERIMENTAL TA FORCE 2

2. PHYSIOLOGICAL AND MATHEMATICAL STEPS TAKEN TO SIMPLIFY THE NEUROMUSCULAR MODEL 5

3. DERIVATION OF THE FREE CALCIUM DYNAMICS 8

4. DERIVATION OF THE CA-TN DYNAMICS 9

5. DERIVATION OF THE DYNAMICS OF MU ACTIVATION 11

1. Derivation of the experimental TA force

Methods

The force F_{cell} (in Volts) recorded by the load cell during the experiments was first converted into an ankle torque T (in N.m) using a constant gain derived from the load cell documentation and the geometry of the dynamometer. It was then assessed with EQ. S1, for the recordings made during the second session where bEMG electrodes were involved, the amount $\Delta T(t)$ of ankle torque taken over the trapezoidal contraction by agonist EHL, EDL and antagonist SOL, GM, and GL muscles. In EQ. S1, $T_i(t)$ is the ankle torque produced by the ith muscle, $\overline{EMG_\iota}(t)$ is the normalized EMG envelope for muscle i computed previously, L is the moment arm between the tendon of muscle i and the ankle joint previously measured from the subject-specific MSK model, $F_{0,i}^M$ is the maximum isometric force of muscle i estimated previously, and f_{FL} is the scaling factor calculated with the FL relationship in EQ.11. $\overline{l_\iota^M}$ is the normalized length of muscle i and was calculated from the subject-muscle-specific l_i^{MT}, $l_{s,i}^T$, and $l_{0,i}^M$ values derived previously, assuming the tendons to be rigid. It was also assumed that the EDL and EHL muscles produced the same electrical activity.

$$\Delta T(t) = T_{EHL}(t) + T_{EDL}(t) - T_{GM}(t) - T_{GL}(t) - T_{SOL}(t)$$
$$T_i(t) = \overline{EMG_\iota}(t) \cdot L_i \cdot F_{0,i}^M \cdot f_{FL}\left(\overline{l_\iota^M}, \overline{EMG_\iota}\right) \qquad \text{EQ. S1}$$
$$\overline{l_\iota^M} = \frac{l_i^{MT} - l_{s,i}^T}{l_{0,i}^M}$$

From EQ. S1, the continuous relationship between the level of load sharing $\Delta T(T)$ and the recorded ankle torque T was computed by fitting the exponential function in EQ. S2 to the $(T;\Delta T)$ cloud of points.

$$\Delta T(T) = a \cdot e^{b \cdot T} \qquad \text{EQ. S2}$$

The fitted $\Delta T(T)$ relationship in EQ. S2 was then applied to the torque $T(t)$ recorded during the first experimental session, for which the level of co-contraction $\Delta T\big(T(t)\big)$ was estimated. The experimental TA muscle force $F_{TA}(t)$ produced during the first experimental session was finally estimated with EQ. S3, where L_{TA} is the moment arm the TA tendon makes with the ankle joint.

$$F_{TA}(t)[N] = \frac{T(t) - \Delta T\big(T(t)\big)}{L_{TA}} \qquad \text{EQ. S3}$$

Results

During the second experimental session, voluntary ankle torques T (black dashed traces in Fig S1) of 5.2, 8.8, and 17.5 Nm were recorded experimentally over the dorsiflexion tasks up to 30%, 50% and 100% MVC, respectively. The results are of the same order of magnitude as the maximum voluntary torque of 22.5 Nm (+28%) calculated with published age-sex-dependent joint angle-torque relationships for ankle dorsiflexion [1]. Using the subject-muscle-specific properties of maximum isometric forces F_0^M and moment arms L reported in Table 2, a maximum torque in ankle dorsiflexion of 41 Nm was theoretically reachable for the participant if the TA, EDL, and EHL muscle produced their maximum force (maximum myoelectric activity at optimal length, i.e., $\overline{EMG} = 1$ and $\overline{l} = 1$ in EQ. S1)

and no co-contraction of the SOL, GM, and GL muscles occurred. In the conditions of the experiment, the TA, EDL, EHL, SOL, GM, and GL worked at 1.18, 0.78, 1.20, 0.60, 0.68, and 0.80 normalized lengths assuming rigid tendons, respectively (Table 2). The myoelectric activity of the EDL/EHL, SOL, GM, and GL muscles reached 27%, 5%, 3%, 6%, and 50%, 16%, 8%, 16%, and 100%, 37%, 30%, 47% of their maximum recorded EMG amplitude, at 30% MVC, 50% MVC, and 100% MVC respectively.

Very similar relationships $\Delta T(T)$, calculated with EQ. S1 and EQ. S2 with the subject-muscle-specific properties reported in Table 2, were obtained for both trapezoidal contractions up to 30% and 50% MVC between the amplitude of measured ankle torque $T(t)$ (black dashed traces in Fig S1) and the amount of ankle torque ΔT taken by the group of agnostic EHL and EDL and antagonist SOL, GM, and GL muscles (blue dotted traces in Fig S1). The average of the two relationships yielded the relationship in EQ. S4 (95% confidence ranges: [-0.1235 ; -0.1170] and [0.3428 ; 0.3479], r^2 = 0.84). EQ. S4 suggests that contractile activities of the antagonist muscles SOL, GM, and GL overtook the ankle torque produced by the agonist EDL and EHL muscles following an exponential tendency with increasing recorded torques T with, for example, $\Delta T = -1.6\,Nm$ at $T = 5.2\,Nm$ (at 30% MVC) and $\Delta T = -7.7\,Nm$ at $T = 8.8\,Nm$ (at 50% MVC).

$$\Delta T(T)[N \cdot m] = -0.1202 \cdot e^{0.3453 \cdot T} \qquad \text{EQ. S4}$$

From EQ. S3, the force developed by the TA was estimated to reach 260 N and 656 N (torques displayed as solid red lines in Fig S1) at 30% and 50% MVC respectively, i.e. 25% and 63% of the TA's maximum isometric force F_0^M (Table 2), which seems reasonable for a participant who aimed to isolate the TA during those contractions. At 30% MVC and 50% MVC, the agonist EDL and EHL muscles collectively produced around 112 N and 215 N of force respectively, i.e., less than half the amount of force produced by the TA muscle despite having more than half the force-generating capacities, which is again consistent with a participant who aimed to isolate the TA during those contractions.

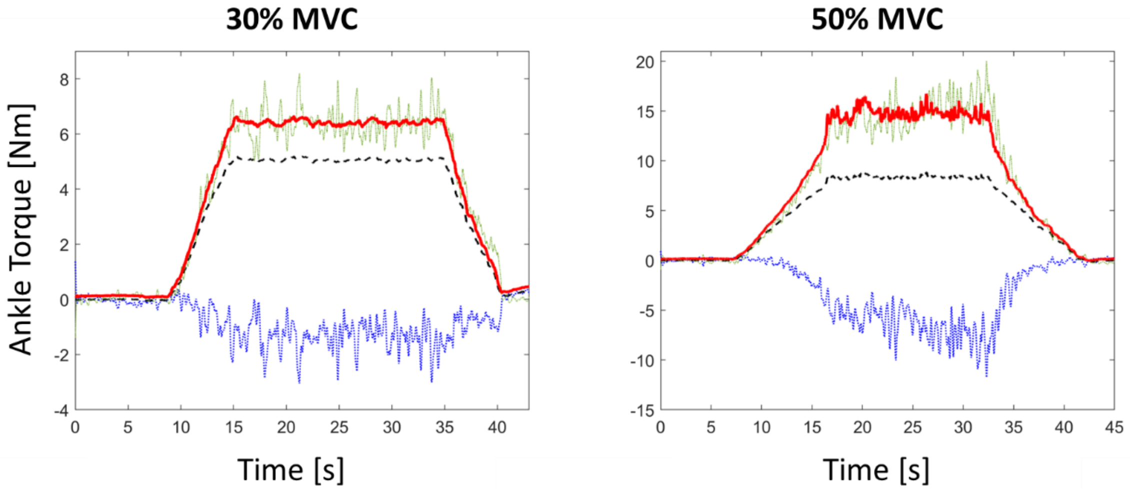

Fig S1: Estimation of the ankle torque developed by the TA muscle (solid red traces) from the recorded ankle torque T (dashed black traces) during the trapezoidal contractions up to 30% (Left) and 50% MVC (right) performed during the second experimental session. The amount of ankle torque ΔT produced by the agonist EDL and EHL and antagonist SOL, GM, and GL muscles (blue dotted traces) was calculated with EQ. S1 using the subject-muscle-specific properties reported in Table 2. ΔT was negative during the contractions, i.e., the agonist and antagonist muscle altogether produced a torque in plantarflexion. The ankle torque developed by the TA

muscle was calculated as $T - \Delta T$ (green dash-dotted traces) and as $T - \Delta T(T)$ (solid red traces), where $\Delta T(T)$ given in EQ. S4 is the continuous relationship fitted to the $(T; \Delta T)$ cloud of points.

2. Physiological and mathematical steps taken to simplify the neuromuscular model

The MN-driven neuromuscular model developed in this study (Fig 1F) consists in a population of n in-parallel FGs, that describes the force-generating activity of the MU pool according to the cascading neuromuscular dynamics of the individual MUs in Fig 3. The FGs are placed in-parallel with a single muscle-scale passive elastic element (PEE) and in-series with a common in-series elastic element (SEE), i.e., the tendon. n is the length of the vector of available input spike trains controlling the model and takes the values N_r or N in this study.

The FGs individually develop the MU active forces $f_k^{MU}, k \in [\![1; n]\!]$, which linearly sum with the PEE force F^{PEE} to yield the total muscle force F^M. Neglecting the low pennation angle between the muscle belly and the tendon of the TA (approximately 11° [2]), which may underestimate F^M by approximately 2%, F^M equals the tendon force F^{SEE} by equilibrium in EQ. S5.

$$F^M = \sum_{k=1}^{n} f_k^{MU} + F^{PEE} = F^{SEE} \qquad \text{EQ. S5}$$

This approach neglects both the geometrical orientation of the individual MUs and the lateral force transmission between MUs, both of which would decrease the total force developed by the MU pool along the tendon if considered.

In the following, all TA quantities are normalized with respect to the MU or whole muscle optimal length or maximum isometric force and are reported with a bar following EQ. S6. In EQ. S6, $f_{k,0}^{MU}$, $l_{k,0}^{MU}$, l_k^{MU}, and $\overline{v_k}$ are the maximum isometric force, the optimal length, the length, and the contraction velocity of the FG of MU k, respectively.

$$\begin{aligned}
\overline{F^{PEE}} &= \frac{F^{PEE}}{F_0^M} & \overline{f_{k,0}^{MU}} &= \frac{f_{k,0}^{MU}}{F_0^M} \\
\overline{F^{SEE}} &= \frac{F^{SEE}}{F_0^M} & \overline{f_k^{MU}} &= \frac{f_k^{MU}}{F_0^M} \\
\overline{l^{MT}} &= \frac{l^{MT}}{l_0^M} & \overline{l_k} &= \frac{l_k^{MU}}{l_{0,k}^{MU}} \\
& & \overline{v_k} &= \frac{d\overline{l_k}}{dt}
\end{aligned} \qquad \text{EQ. S6}$$

The individual normalized MU forces $\overline{f_k^{MU}}$ produced by the MU FGs were modelled in EQ. S7 to be the linear product of the MU normalized maximum isometric force $\overline{f_{k,0}^{MU}}$, the MU active state a_k, the MU normalized force-length (FL) scaling factor f_{FL}, and the MU normalized force-velocity (FV) scaling factor f_{FV}.

$$\overline{f_k^{MU}}\left(a_k, \overline{l_k}, \bar{v}_k\right) = \overline{f_{k,0}^{MU}} \cdot a_k\left(\overline{l_k}\right) \cdot f_{FL}\left(a_k, \overline{l_k}\right) \cdot f_{FV}\left(a_k, \overline{l_k}, \bar{v}_k\right) \qquad \text{EQ. S7}$$

Inserting EQ. S7 into EQ. S5, it yields EQ. S8.

$$\overline{F^{SEE}} - \overline{F^{PEE}} = \sum_{k=1}^{n} \overline{f_{k,0}^{MU}} \cdot \left(a_k(\overline{l_k}) \cdot f_{FL}(a_k, \overline{l_k}) \cdot f_{FV}(a_k, \overline{l_k}, \overline{v}_k)\right) \qquad \text{EQ. S8}$$

EQ. S8 cannot be solved without knowing, for each MU, the value of its individual fibre architecture (optimal fibre length, fibre length), which implies knowing their spatial distribution, at all frames and active state. The muscle quantities are currently unfeasible to measure and therefore we implemented a number of simplifications displayed in Fig S2 and detailed in the following. First, it was assumed that all the MUs in the MU pool shared the same optimal MU length l_0^{MU}, which was taken to be the subject-specific muscle optimal length l_0^M derived previously in the main manuscript in EQ. 2. Second, expecting the MU optimal lengths $l_{0,k}^{MU}$ to be related to the MU lengths l_k^{MU}, all the MUs in the MU pool were assigned a common (normalized) length $\bar{l} = \frac{l^{MU}}{l_0^M}$ and by extension a common normalized contraction velocity $\bar{v}$, as $\bar{v} = \frac{d\bar{l}}{dt}$. Third, to isolate $\bar{v} = \frac{d\bar{l}}{dt}$ and rearrange EQ. S8 as a differential equation of the common normalized MU length $\bar{l}$, the MU FV relationship f_{FV} was simplified to be independent from the individual MU active state a_k, which yielded EQ. S9.

$$\bar{v} = \frac{d\bar{l}}{dt} = f_{FV}^{-1}\left(\frac{\overline{F^{SEE}} - \overline{F^{PEE}}}{\sum_k \overline{f_{k,0}^{MU}} \cdot \left(a_k(\bar{l}) \cdot f_{FL}(a_k, \bar{l})\right)}\right) \qquad \text{EQ. S9}$$

Although EQ. S9 can be solved numerically for $\bar{l}$, the computational cost and the complexity of the model were further reduced by first assuming the TA tendon to be rigid {4}. As $\frac{l_s^T}{l_0^M} = 3.5$ for the participant's TA muscle, this assumption was judged acceptable for low-to-medium TA isometric contractions, considering that $\frac{l_s^T}{l_0^M} = 1$ describes a 'very stiff' tendon in the literature [3]. With this assumption, the tendon length is constant and equals the tendon slack length [4] $l^{SEE} = l_s^T$. As l^{MT} is also constant during isometric contractions, so is the common normalized MU length $\bar{l}$, in which case $f_{FV} = 1$. Second, because $l^{MT} = l_s^T + 1.16 \cdot l_0^M$ for the segmented TA muscle in the conditions of the experiment (see Results Section for the subject-specific values calculated with EQ. 2), it was finally approximated {5} that the FGs worked at the common normalized length $\bar{l} = 1.16$, in which case $\overline{F^{PEE}} = 0.02 \approx 0$, according to EQ. S10 with $k_1 = 5$ and $k_2 = 0.6$ for human young adults [5].

$$\overline{F^{PEE}}(\bar{l}) = \begin{cases} 0 & ; \quad \bar{l} < 1 \\ \dfrac{e^{\frac{k_1}{k_2}(\bar{l}-1)} - 1}{e^{k_1} - 1} & ; \quad \bar{l} \geq 1 \end{cases} \qquad \text{EQ. S10}$$

With these five key simplifications {1-5}, the PEE and SEE (in grey in Fig 1F) were neglected and the rheological structure was reduced to the cohort of in-parallel MU actuators (in black in Fig 1F), the dynamics of which reduced to EQ. S10 in the main text of the manuscript

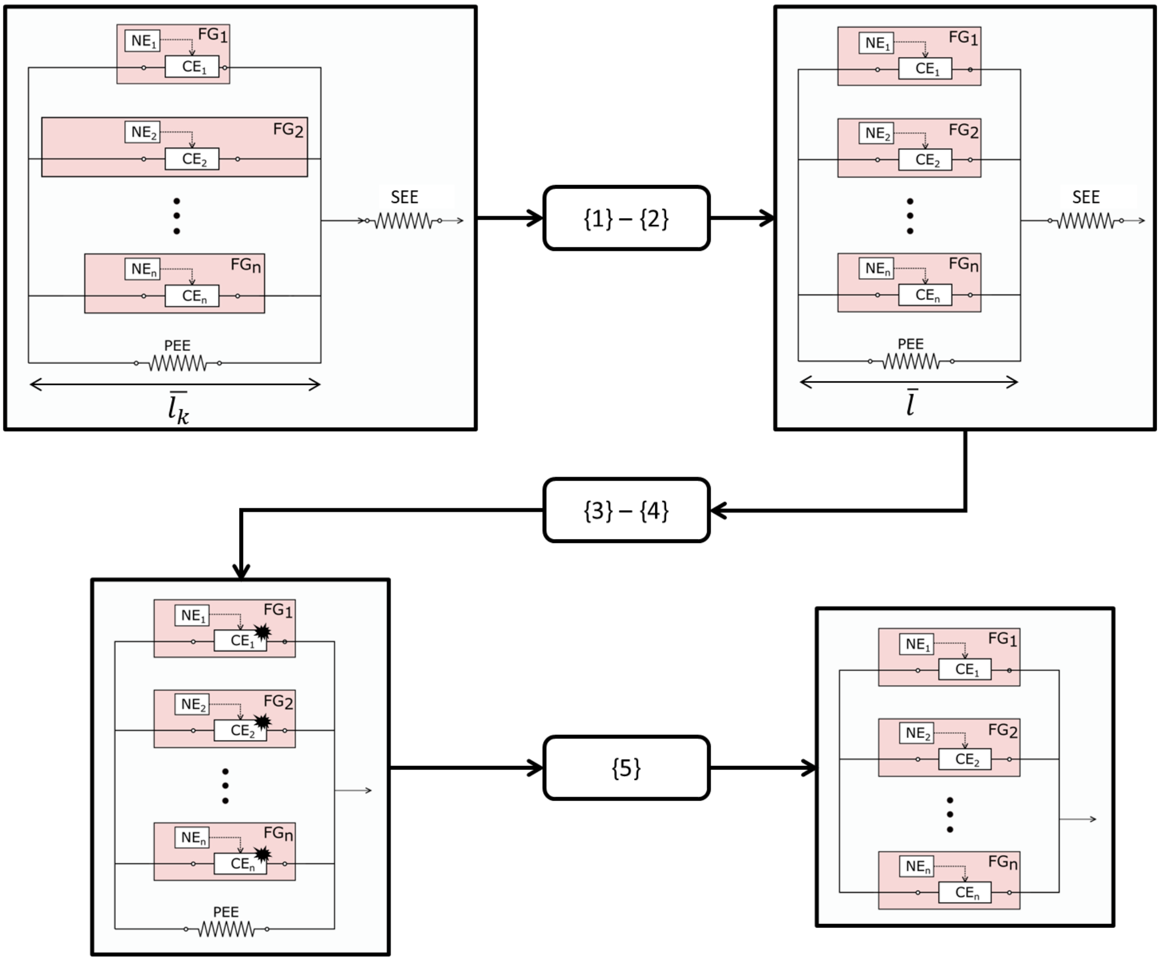

Fig S2: Step-by-step simplification of the MN-driven model. After neglecting the pennation angle between the TA muscle and tendon, the MUs are first assumed to share a common optimal length, which is the whole muscle's optimal length l_0^M {1} and a common normalized length $\bar{l}$ {2}. Then, the MUs' FV relationships are simplified to be activation-dependent {3}, before the tendon is assumed rigid {4}. Consequently, the SEE is neglected, the muscle contracts isometrically, and the FV relationship is neglected. Finally, based on subject-specific MSK measurements, it yields $\bar{l} = 1.16$, in which case the PEE develops negligible force and is neglected {5}.

3. Derivation of the free Calcium dynamics

Despite some disagreements [6], it has been repeatedly observed in *in vitro* experiments on amphibian fibres from the semitendinosus, iliofibularis and TA muscles [7-10] that the peak value of the calcium concentration nonlineary varies with the sarcomere length l^s. The experimental data provided by these studies was manually digitized and normalized to the highest peak $[Ca^{2+}]$ value retrieved in each study and to the optimal sarcomere length l_0^s $\left(\bar{l} = \frac{l^s}{l_0^s}\right)$, which is typically $2.1\mu m$ in these amphibian muscles. The piecewise linear trendline $f_1(\bar{l})$ given in EQ. 15 was fitted to the normalized data, which reveals that the peak $[Ca^{2+}]$ value is obtained between 1.15 and 1.30 l_0^s.

The $f_2(\bar{l})$ scaling factor described in EQ. 16 was obtained following a similar approach and models the linear increase in τ_d with longer normalized fibre lengths $\bar{l}$ above $1.15 l_0^s$ observed experimentally [10].

In the literature of Hill-type muscle models, the coefficients scaling the mathematical equations describing the calcium transients (b_1, b_2, b_3 in EQ. 14) are typically tuned to match dated experimental observations of calcium waves obtained *in vitro* from amphibian muscle fibres in low-temperature baths [11]. Yet, the calcium dynamics in the sarcoplasm are strongly species-dependent, which transients twice longer in amphibians than in mammals [12], and temperature-dependent [7,10,13-23]. Moreover, these dated experimental observations were typically obtained with chemical indicators (murexide, aequorin and arsenazo III for example) that have a high affinity to components other than calcium ions in the myoplasm, such as Mg ions, and report calcium twitches of too low peak values and delayed transients. Because of these limitations, rather than considering the values proposed in Hatze's work [24] for the b_1, b_2, b_3 coefficients, which were obtained in frog fibres at 9°C with the aequorin indicator, the b_1, b_2, b_3 coefficients were here tuned to match the experimental data from two experimental studies [12,25], where the experimental $[Ca^{2+}]$ waves were measured at 28-35°C in rodent fibres with the reliable [10,12,15,19,21,25] mag-fura-2 indicator. Lacking human data in the literature, it was believed that the calcium dynamics in rodents at 35°C were the most representative for the human species at body temperature. Because both studies [12,25] performed their experiments at the same sarcomere length $l^s = 3.8\mu m \approx 1.6 l_0^s$ [26], the length-dependent scaling factors were set to $f_1(1.6) = 0.82$ and $f_2(1.6) = 0.82$ when tuning the b_1, b_2, b_3 coefficients.

4. Derivation of the Ca-Tn dynamics

As previously reported [27], cross-bridge formation increases the Ca^{2+} affinity of troponin and slows down Ca^{2+} detachment from troponin. When triggered by a single AP, the decay rate constant τ_d of the CaTn twitch therefore strongly decreases as the filamentary overlap decreases. In other terms, when $\bar{l}$ increases above 1.0, CaTn decays more rapidly. Consequently, the mag-2-fura measurements of CaTn, which overlook this affinity to cross-bridge formation, are not reliable at the sarcomere lengths for which filamentary overlap occurs, and the trajectory of the intensity of the meridional reflection from troponin at 1/38.5nm^{-1} must be used instead to measure CaTn [27].

In [27], experiments were performed in frogs at 16°C, in muscles of dominantly fast fibres at sarcomere lengths $l^s = 1.9l_0^s$ (nonoverlap state) and $l^s = 1.3l_0^s$ (overlap state). The experimental CaTn time traces were digitized and the t_{tp} and τ_d values were measured at both lengths l^s. To obtain CaTn values for rodents and slow fibres, the available experimental data (frog, fast fibre, 16°C) was extrapolated, in first approximation, to rodent data at 35°C in slow fibres by using the same species- temperature- and fibre type-scaling factors as used with the $[Ca^{2+}]$ transients previously investigated. In three steps, the t_{tp} and τ_d frog quantities were therefore gradually multiplied by 0.5, 0.44, and 2.5. Because the t_{tp} and τ_d values, initially measured at $l^s = 1.9l_0^s$ and $l^s = 1.3l_0^s$, linearly depend on the number of formed cross-bridges, piecewise linear length-dependent relationships $t_{tp}(\bar{l})$ and $\tau_d(\bar{l})$ were inferred (Table S1) from a typical piecewise-linear force-length shape [26] corrected for rodent optimal sarcomere lengths $l_0^s = 2.4\mu m$. Finally, the CaTn peak-to-peak amplitude was set to $120\mu M$ and $220\mu M$ for slow and fast fibres respectively [25,28,29].

As before, the $f_3(\bar{l}), f_4(\bar{l}), f_5(\bar{l})$ length-dependent scaling factors and the c_1, c_2, P_0 parameters, respectively reported in Table S1 and Table 1, were tuned so that the output of the differential equation in EQ. 17 matched the scaled experimental length-dependent t_{tp} and τ_d, and A_t quantities in the first column of Table S1 with less than 5% error.

Table S1: (Left) Typical t_{tp}, τ_d and A_t values of the CaTn concentration twitch in rodent fast and slow fibres, and their length-dependencies, inferred from experimental amphibian data. (Right) Description of the length-dependent scaling factors in EQ. 17.

$[Ca - Tn]$ metrics	Length-dependent scaling factors
$t_{tp,fast}(\bar{l}) = \begin{cases} 6ms \;\; if\ \bar{l} < 0.7 \\ 2 + 5\bar{l} \;\; if\ \bar{l} \in [0.7; 0.97] \\ 7ms\ if\ \bar{l} \in [0.97; 1.02] \\ 12 - 5\bar{l} \;\; if\ \bar{l} \in [1.02; 1.6] \\ 4ms \;\; if\ \bar{l} > 1.6 \end{cases}$	$\begin{cases} f_3(\bar{l}) = 0.33 & if\ \bar{l} \leq 0.7 \\ f_3(\bar{l}) = 0.33 + 2.47(\bar{l} - 0.75) & if\ \bar{l} \leq 0.97 \\ f_3(\bar{l}) = 1.0 & if\ 0.97 \leq \bar{l} \leq 1.02 \\ f_3(\bar{l}) = 8.9 - 11.73\bar{l} + 3.90\,\bar{l}^2 & if\ 1.02 \leq \bar{l} \leq 1.6 \\ f_3(\bar{l}) = 0.125 & if\ \bar{l} > 1.6 \end{cases}$
$\tau_{d,fast}(\bar{l}) = \begin{cases} 15ms \;\; if\ \bar{l} < 0.7 \\ -1 + 23\bar{l} \;\; if\ \bar{l} \in [0.7; 0.97] \\ 21ms\ if\ \bar{l} \in [0.97; 1.02] \\ 44 - 23\bar{l} \;\; if\ \bar{l} \in [1.02; 1.6] \\ 8ms \;\; if\ \bar{l} > 1.6 \end{cases}$	$\begin{cases} f_5(\bar{l}) = 0.75 & if\ \bar{l} \leq 0.7 \\ f_5(\bar{l}) = 0.75 + 0.94(\bar{l} - 0.75) & if\ \bar{l} \leq 0.97 \\ f_5(\bar{l}) = 1.0 & if\ 0.97 \leq \bar{l} \leq 1.02 \\ f_5(\bar{l}) = 0.9 + 0.84\bar{l} - 0.75\,\bar{l}^2 & if\ 1.02 \leq \bar{l} \leq 1.6 \\ f_5(\bar{l}) = 0.34 & if\ \bar{l} > 1.6 \end{cases}$
$A_{t,fast} = 220\mu M$	$\begin{cases} f_4(\bar{l}) = 1.09 & if\ \bar{l} \leq 0.7 \\ f_4(\bar{l}) = 1.09 - 0.32(\bar{l} - 0.75) & if\ \bar{l} \leq 0.97 \\ f_4(\bar{l}) = 1.0 & if\ 0.97 \leq \bar{l} \leq 1.02 \\ f_4(\bar{l}) = -4.4 + 8.24\bar{l} - 2.84\,\bar{l}^2 & if\ 1.02 \leq \bar{l} \leq 1.6 \\ f_4(\bar{l}) = 1.46 & if\ \bar{l} > 1.6 \end{cases}$
$t_{tp,slow}(\bar{l}) = \begin{cases} 11ms \;\; if\ \bar{l} < 0.7 \\ 4 + 10\bar{l} \;\; if\ \bar{l} \in [0.7; 0.97] \\ 14ms\ if\ \bar{l} \in [0.97; 1.02] \\ 24 - 10\bar{l} \;\; if\ \bar{l} \in [1.02; 1.6] \\ 8ms \;\; if\ \bar{l} > 1.6 \end{cases}$	$\begin{cases} f_3(\bar{l}) = 0.75 & if\ \bar{l} \leq 0.7 \\ f_3(\bar{l}) = 0.75 + 0.93(\bar{l} - 0.75) & if\ \bar{l} \leq 0.97 \\ f_3(\bar{l}) = 1.0 & if\ 0.97 \leq \bar{l} \leq 1.02 \\ f_3(\bar{l}) = 1.2 - 0.01\bar{l} - 0.22\,\bar{l}^2 & if\ 1.02 \leq \bar{l} \leq 1.6 \\ f_3(\bar{l}) = 0.67 & if\ \bar{l} > 1.6 \end{cases}$
$\tau_{d,slow}(\bar{l}) = \begin{cases} 38ms \;\; if\ \bar{l} < 0.7 \\ -3 + 57\bar{l} \;\; if\ \bar{l} \in [0.7; 0.97] \\ 52ms\ if\ \bar{l} \in [0.97; 1.02] \\ 110 - 57\bar{l} \;\; if\ \bar{l} \in [1.02; 1.6] \\ 19ms \;\; if\ \bar{l} > 1.6 \end{cases}$	$\begin{cases} f_5(\bar{l}) = 0.66 & if\ \bar{l} \leq 0.7 \\ f_5(\bar{l}) = 0.66 + 1.27(\bar{l} - 0.75) & if\ \bar{l} \leq 0.97 \\ f_5(\bar{l}) = 1.0 & if\ 0.97 \leq \bar{l} \leq 1.02 \\ f_5(\bar{l}) = 4.2 - 4.28\bar{l} - 1.11\,\bar{l}^2 & if\ 1.02 \leq \bar{l} \leq 1.6 \\ f_5(\bar{l}) = 0.21 & if\ \bar{l} > 1.6 \end{cases}$
$A_{t,slow} = 120\mu M$	$\begin{cases} f_4(\bar{l}) = 1.06 & if\ \bar{l} \leq 0.7 \\ f_4(\bar{l}) = 1.06 - 0.23(\bar{l} - 0.75) & if\ \bar{l} \leq 0.97 \\ f_4(\bar{l}) = 1.0 & if\ 0.97 \leq \bar{l} \leq 1.02 \\ f_4(\bar{l}) = 4.5 - 5.09\bar{l} + 1.62\,\bar{l}^2 & if\ 1.02 \leq \bar{l} \leq 1.6 \\ f_4(\bar{l}) = 0.52 & if\ \bar{l} > 1.6 \end{cases}$

5. Derivation of the dynamics of MU activation

The d_i coefficients in EQ. 18 were tuned so that the predicted MU active state produced by a CaTn twitch matched the normalized experimental twitch torque or force $\overline{f^{tw}}$ recorded in individual human MUs in the TA muscle [30,32]. Although the experimental $\overline{f^{tw}}$ also accounts for mechanical dynamics other than the activation dynamics, such as the muscle-tendon interplay or muscle co-contraction and may not be a precise indicator of the MU active state, it remains the only available metric for a in the human literature. Because the physiological length-dependency of the cross-bridge attachment dynamics at known Ca-Tn concentration P is unclear in the literature, the d_1, d_2 and d_3 parameters were modelled constant at all lengths and were calibrated at optimal length $\bar{l} = 1.0$. Although it is debated in the literature, as previously reviewed [4], whether the MU type is correlated to t_{tp} and $t_{0.5}$, it was assumed for simplicity that the slow-type MUs had the longest time-to-peak and half relaxation time values in the MU pool. Considering that 12% of the MUs are of the fast type in the MU pool (Fig 2D), and assuming that 12% of the MUs identified in the aforementioned experimental TA literature were of the fast type, it was derived from the t_{tp} and $t_{0.5}$ bar plot distributions in these studies that the slow and fast type MUs respectively had t_{tp} values in the $[35 - 90]ms$ and $[20 - 35]ms$ ranges, of weighted average 50ms and 30ms, and $t_{0.5}$ values in the $[50 - 90]ms$ and $[40 - 50]ms$ ranges, of weighted average 65 and 42ms. Finally, d_1 was tuned so that the active state reached 1.0 at 50 Hz stimulation, as firing frequencies above 50Hz were never reported in the human literature.

REFERENCES

1. Anderson DE, Madigan ML, Nussbaum MA. Maximum voluntary joint torque as a function of joint angle and angular velocity: Model development and application to the lower limb. J.Biomech. 2007 40:3105-3113.
2. Rajagopal A, Dembia CL, DeMers MS, Delp DD, Hicks JL, Delp SL. Full-body musculoskeletal model for muscle-driven simulation of human gait. TBME 2016, 63:2068-2079.
3. Zajac Felix E. Muscle and tendon: Properties, models, scaling, and application to biomechanics and motor control. Critical reviews in biomedical engineering 1989, 17:359-411.
4. Millard M, Uchida T, Seth A, Delp SL. Flexing computational muscle: Modeling and simulation of musculotendon dynamics. Journal of biomechanical engineering 2013, 135:021005.
5. Thelen DG. Adjustment of muscle mechanics model parameters to simulate dynamic contractions in older adults. J Biomech Eng 2003, 125:70-77.
6. Haugen P. Calcium transients in skeletal muscle fibres under isometric conditions and during and after a quick stretch. Journal of Muscle Research & Cell Motility 1991, 12:566-578.
7. Blinks JR, Rüdel R, Taylor SR. Calcium transients in isolated amphibian skeletal muscle fibres: Detection with aequorin. J.Physiol. 1978, 277:291-323.
8. Baylor SM, Chandler WK, Marshall MW. Sarcoplasmic reticulum calcium release in frog skeletal muscle fibres estimated from arsenazo III calcium transients. J.Physiol. 1983, 344:625-666.
9. Close RI, Lännergren JI. Arsenazo III calcium transients and latency relaxation in frog skeletal muscle fibres at different sarcomere lengths. J.Physiol. 1984, 355:323-344.
10. Konishi M, Hollingworth S, Harkins AB, Baylor SM. Myoplasmic calcium transients in intact frog skeletal muscle fibers monitored with the fluorescent indicator furaptra. J.Gen.Physiol. 1991, 97:271-301.
11. Caillet AH, Phillips AT, Carty C, Farina D, Modenese L. Hill-type computational models of muscle-tendon actuators: A systematic review. bioRxiv 2022, doi: https://doi.org/10.1101/2022.10.14.512218.
12. Hollingworth S, Zhao M, Baylor SM. The amplitude and time course of the myoplasmic free [Ca2] transient in fast-twitch fibers of mouse muscle. J.Gen.Physiol. 1996, 108:455-469.
13. Miledi R, Parker I, Zhu PH. Calcium transients evoked by action potentials in frog twitch muscle fibres. J.Physiol. 1982, 333:655-679.
14. Eusebi F, Miledi R, Takahashi T. Aequorin-calcium transients in frog twitch muscle fibres. J.Physiol. 1983, 340:91-106.
15. Hirota A, Chandler WK, Southwick PL, Waggoner AS. Calcium signals recorded from two new purpurate indicators inside frog cut twitch fibers. J.Gen.Physiol. 1989, 94:597-631.
16. Raju B, Murphy E, Levy LA, Hall RD, London RE. A fluorescent indicator for measuring cytosolic free magnesium. American Journal of Physiology-Cell Physiology 1989 ,256:C540-C548.
17. Delbono O, Stefani E. Calcium transients in single mammalian skeletal muscle fibres. J.Physiol. 1993, 463:689-707.
18. Delbono O, O'rourke KS, Ettinger WH. Excitation-calcium release uncoupling in aged single human skeletal muscle fibers. J.Membr.Biol. 1995, 148:211-222.

19. Zhao M, Hollingworth S, Baylor SM. Properties of tri-and tetracarboxylate Ca2 indicators in frog skeletal muscle fibers. Biophys.J. 1996, 70:896-916.
20. Capote J, Bolaños P, Schuhmeier RP, Melzer W, Caputo C. Calcium transients in developing mouse skeletal muscle fibres. J.Physiol. 2005, 564:451-464.
21. Hollingworth S, Zeiger U, Baylor SM. Comparison of the myoplasmic calcium transient elicited by an action potential in intact fibres of mdx and normal mice. J.Physiol. 2008, 586:5063-5075.
22. Baylor SM, Hollingworth S. Calcium indicators and calcium signalling in skeletal muscle fibres during excitation–contraction coupling. Prog.Biophys.Mol.Biol. 2011, 105:162-179.
23. Hollingworth S, Kim MM, Baylor SM. Measurement and simulation of myoplasmic calcium transients in mouse slow-twitch muscle fibres. J.Physiol. 2012, 590:575-594.
24. Hatze H. A myocybernetic control model of skeletal muscle. Biological cybernetics 1977, 25:103-119.
25. Baylor SM, Hollingworth S. Sarcoplasmic reticulum calcium release compared in slow-twitch and fast-twitch fibres of mouse muscle. J.Physiol. 2003, 551:125-138.
26. Rassier DE, MacIntosh BR, Herzog W. Length dependence of active force production in skeletal muscle. J.Appl.Physiol. 1999, 86:1445-1457.
27. Matsuo T, Iwamoto H, Yagi N. Monitoring the structural behavior of troponin and myoplasmic free Ca2 concentration during twitch of frog skeletal muscle. Biophys.J. 2010, 99:193-200.
28. Baylor SM, Hollingworth S. Simulation of Ca2 movements within the sarcomere of fast-twitch mouse fibers stimulated by action potentials. J.Gen.Physiol. 2007, 130:283-302.
29. Baylor SM, Hollingworth S. Intracellular calcium movements during excitation–contraction coupling in mammalian slow-twitch and fast-twitch muscle fibers. J.Gen.Physiol. 2012, 139:261-272.
30. Andreassen S, Arendt-Nielsen L. Muscle fibre conduction velocity in motor units of the human anterior tibial muscle: A new size principle parameter. J. Physiol. 1987, 391:561-571.
31. Van Cutsem M, Feiereisen P, Duchateau J, Hainaut K. Mechanical properties and behaviour of motor units in the tibialis anterior during voluntary contractions. Can. J. Appl. Physiol. 1997, 22:585-597.
32. Van Cutsem M, Duchateau J, Hainaut K. Changes in single motor unit behaviour contribute to the increase in contraction speed after dynamic training in humans. J.Physiol. 1998, 513:295-305.
