# Supplementary material for: Motoneuron-driven computational muscle modelling with motor unit resolution and subject-specific musculoskeletal anatomy
Source: PLoS Comput Biol. 2023 Dec 7;19(12):e1011606. doi: 10.1371/journal.pcbi.1011606 (PMC10729998; doi:10.1371/journal.pcbi.1011606)
Supplement: Attachment — Submitted filename: 2023-09-29_Response to reviewers_LM.docx [file pcbi.1011606.s002.docx]

**Response to Reviewers’ and Editor’s Comments:**

Throughout the document, the authors’ responses are in red while the original reviewers’ and editor’s comments are in black.

As with all papers reviewed by the journal, your manuscript was reviewed by members of the editorial board and by several independent reviewers. The reviewers appreciated the attention to an important topic. Based on the reviews, we are likely to accept this manuscript for publication, providing that you modify the manuscript according to the review recommendations.

Please note in particular that, although both reviewers appreciated the thorough approach in the presented muscle model, they felt that there should be more discussion of how it fits into the context of existing models. Under what circumstances would this model (rather than a simpler one) be most applicable? How do the results compare to the classic single-input Hill-type model and what is the relative computational cost?

We thank the editor and the reviewers for their positive feedback and appreciation of our work. We revised the manuscript according to the provided recommendations by carefully addressing each of the reviewers’ comments, all of which focused on the Discussion Section.

In the first version of the manuscript, we had assessed in detail the novelty that our MN-driven model of MU pool brings to the field of neuromuscular modelling, supported by a recent systematic review (Caillet et al., 2022a). In this revised version of the manuscript, we further analyse how our model fits into the context of existing models by discussing in detail:

- The circumstances where our model, rather than a simpler one, would be most applicable. This discussion now lies in a new sub-section of the Discussion ‘Applications and future perspectives’ (P45, L1068). Several specific applications where MN-driven models of MU pools are more advantageous than classic single-actuator models are now presented and discussed in the scope of the fields of human-machine interfacing, biomechanics, neurophysiology, muscle physiology, and muscle neuromechanics.
- The comparative performance of our model with classic single-input Hill-type models (P39, L877). To do so, we have reviewed the literature that performed comparative assessments between single-actuator and MU pool models, and we discussed our model in the light of these studies. As now expressed in the manuscript, this comparative literature is scarce, and the field requires a yet inexistant systematic comparison of these types of models. We are currently conducting such an analysis that we aim to make available in a separate manuscript; considering the length and density of the current manuscript, integrating a complex additional analysis of this type to the present study would make the manuscript longer and more difficult to follow for the reader.
- The relative computational cost of our model with classic single-input Hill-type models (P47, L1132.

Please note that the two sub-sections ‘Model scaling’ and ‘Interfacing the fields of motor control and MSK modelling’ have been merged into the new sub-section ‘Interfacing the fields of subject-specific motor control, neuromuscular modelling, and MSK modelling’ in the Discussion Section to maintain a clear narrative and overall clarity in this long manuscript after the reviewers’ comments were integrated.

**Reviewer #1:**

The manuscript presents the detailed development and validation of a motoneuron driven, physiologically accurate, muscle model. Subject and muscle specific parameters were derived from medical imaging and literature data to generate an accurate representation of the individual neuromuscular anatomy and physiology. Motoneuron spike trains were experimentally derived from measurement of the tibialis anterior muscles during submaximal voluntary isometric contraction, and used to drive and validate the proposed model. Three different grid configurations, with varying density of HDEMG, were evaluated to assess the effect of experimental recording quality on the TA force prediction. The verification of the modelling pipeline was detailed and provided convincing results that supported the conclusions of the manuscript.

Overall, this paper is carefully written and a wonderful example of how a computational model should be developed and validated. Every modelling choice was planned, justified, and supported by literature. I could not fault any part of the modelling that was not already detailed in the limitations. The well organised equations also provide an excellent summary of much of the cited literature, which was needed. Finally, I truly appreciated that the authors provided the modelling code, which will surely boost future impact.

We thank the reviewer for their positive feedback and appreciation of the systematic and careful approach we took to develop our open-source model.

Generic comments

1. While the paper is technically excellent, I believe the parts of the discussions related to future applications were a bit vague and generic. The number of required parameters, the challenges of data acquisition, and the inability to function in dynamic conditions might limit the future use of the proposed model for most biomechanics applications. Overall, I would appreciate to see the discussion extended to highlight and detail how future applications or scientific investigations could truly leverage this model.

We understand the concerns raised by the reviewer. The reasons for the complexity of our model were not sufficiently and clearly justified in light of specific applications. We addressed this comment in the revised version of the manuscript by gathering all previous scattered occurrences related to that discussion in a new dedicated subsection of the Discussion section entitled ‘Applications and future perspectives’ (P45, L1068). In this new section, the discussion is now enriched with examples of specific applications where MN-driven models of MU pool would be more adequate than a standard single-actuator bipolar EMG (bEMG)-driven model. Specific examples were given in the fields of human-machine interfacing, biomechanics, neurophysiology, muscle physiology, and muscle neuromechanics. Then, as suggested by the reviewer, we discussed in detail how some specific limitations brought by the additional complexity of our model (like computational load, challenges in data acquisition, static conditions…) might limit the future use of the proposed model, and how current research is addressing those challenges.

**P45, L1068:**

*“Applications and future perspectives*

*The MN-driven multi-MU model proposed in this study comprehensively describes the recruitment and firing strategy of the MU pool during human voluntary muscle contractions. For these reasons, this detailed model finds applications in answering scientific questions where the lump dynamics of the classic single-actuator bEMG-driven Hill-type models provide limited help.*

*In the study of the human muscle architecture, by combining the aforementioned mapping of the MU pool in volumetric muscle representations with recently developed ultrasound measurement techniques for tracking MU twitches and mechanical properties (Lubel et al., 2022; Lubel et al., 2023), one could gain insights into the distribution of MU territories within human muscles, which remains an open question. The volumetric mapping of MUs also provides a convenient framework for modelling the transversal mechanical interactions between MUs (Herzog, 2019), and the resulting force-varying load path within the muscle tissue, that results in a nonlinear summation of the individual MU forces.*

*In the field of neurophysiology, the MN-driven model of MU population proposed in this study is suited for integrating MN synergies in simulations. Contrary to single-input Hill-type models that lump the dynamics of synaptic input, MN recruitment, and MN discharge into a single phenomenological signal, the MU pool modelled in Figure 1 can be divided into functional clusters and receive different common inputs to reduce the dimensionality of the control (Hug et al., 2023). For example, in multi-muscle MSK models with MU-actuated volumetric representations of muscles, the model proposed in Figure 1 is also suited for the investigation of neural synergies between muscles, where MN clusters span across muscles (Hug et al., 2023), with strategies specific to the muscle groups (Del Vecchio et al., 2023).*

*In the study of human muscle contraction dynamics, the model developed in this study provides a credible window onto the distribution of the excitation-contraction dynamics of individual MUs across the MU pool (Figure 4) that cannot be measured in human in vivo, and advances our understanding of the muscle-specific neuromechanical strategies for muscle force generation. For example, Figure 4 suggests that, at 30% MVC, the highest-threshold recruited MUs with the largest force-generating capacities are not those producing the highest forces within the recruited pool due to low activation states explained by the onion skin theory (Figure 8A, D). As the model integrates realistic descriptions of the MU-specific firing, recruitment, excitation, and activation dynamics responsible for muscle force generation, it also provides a convenient framework for integrating and investigating MU-related mechanisms, such as the effect of fatigue on the MU firing and recruitment strategies (Taylor et al., 2016) or other fatigue-induced changes in MU activation dynamics as already proposed in single-MU models (Callahan 2016, Shorten 2007).*

*However, some currently investigated limitations can prevent the proposed MN-driven MU-based model from being readily applicable to the aforementioned scientific questions. First, although the pipeline in Figure 1 is fully suited for dynamic contractions, the study proposed here was constrained to isometric tasks due to current experimental limitations in the decomposition of HDEMG signals into MU spike trains during motion. New computational approaches are emerging to identify individual spike trains in quasi-static (Oliveira & Negro, 2021) and dynamic (Glaser & Holobar, 2018) tasks, where 7-20 MUs can be currently identified. Second, data acquisition remains challenging and time-consuming. As opposed to straightforward bEMG recording and filtering, the pipeline in Figure 1 requires decomposing HDEMG signals and manually editing the identified spike trains editing, while taking precautions to identify the full spectrum of discharging motor units for accurate predictions (Figure 6), as discussed. These challenges should be addressed with the rapid emergence of guidelines for manufacturers on HDEMG grid design (Caillet et al., 2023), open-source tools for automatic HDEMG decomposition and spike trains edition (Avrillon et al., 2023), automated spike train identification approaches based on machine learning (Clarke et al., 2020) and blind-source separation methods (Rossato et al., 2023), and MU pool reconstruction methods (Caillet et al., 2022b).*

*Finally, with four ODEs to solve at each time step for each of the modelled motor units, the model proposed in this study is currently computationally too expensive for real-time applications, as opposed to the classic single-input Hill-type actuators, which usually include zero to two ODEs. For the simulation of a 40s-long muscle contraction using a standard laptop (RAM: 12 GB, CPU: one Intel Core i7-1165G7 2.80 GHz), the pipeline in Figure 1 implemented in Python ran in 18 minutes for a MU pool of* $N_{r}=16$ *MUs (Figure 8A, dotted trace), in 63 minutes for* $N_{r}=81$ *MUs (Figure 8C, dotted trace), and in 240 to 300 minutes for the complete pool of* $N=400$ *MUs (Figure 8, red traces), that additionally required preliminary MU pool reconstruction. For comparison, a single-actuator rigid-tendon Hill-type model (Millard et al., 2013) ran in less than a minute on the same machine for the same 40s-long trapezoidal isometric contraction where the HDEMG signals were averaged and normalized to MCV signals. Besides implementations of the model in a faster compiled programming language (for example C++) and using better performing machines, the computational speed could be drastically increased by using parallel computing to solve the independent MU dynamics, by considering smaller reconstructed populations of 50 MUs without loss of accuracy in the estimated neural drive to muscle, as previously discussed (Caillet et al., 2022b), or by, for example, simplifying the current model of MU excitation and activation dynamics with analytical descriptions of the fibre APs and calcium transients instead of the two 2nd-order ODEs in EQ. 13 and EQ. 14.”*

2. As the focus of the paper was on muscle force, one is left wondering how this modelling approach would compare to a classic single-fibre Hill-type bEMG-driven muscle model. An additional analysis of this type would be very useful.

We agree with the reviewer that benchmarking the prediction accuracy of our model against classic single-actuator bEMG-driven models is important. This is the reason why we have been preparing a separate study in which we are undertaking a systematic comparative assessment of MU pool and simpler single-actuator models. To address the reviewer’s comment, we added a new paragraph (P39, L877) to the section where we discuss how our model fits into the context of existing models. In this paragraph, we now review the literature that provides comparative assessments of MU pool models against single-actuator muscle-scale models. We come to the conclusion that MU pool models are expected to provide more accurate predictions of muscle force in the frequency domain, while the literature indicates that similar performance can be expected in the time domain. However, other evidence suggest that MU pool models might be more accurate in the time domain in specific contraction tasks where different neural strategies are involved.

It is also recalled in this new paragraph that the proposed MU pool model is not limited to predictions of muscle force from individual motoneuronal activity. It also provides a window into the muscle’s internal dynamics that cannot be observed *in vivo*, and finds dedicated applications as addressed in the reviewer’s previous comment.

As now discussed in this new paragraph, the literature that performs comparative analysis of MU pool and single-actuator models is very scarce. Therefore, the field requires a systematic comparative study that involves a variety of contraction tasks, types of inputs, and variants of Hill-type models to derive robust conclusions. Considering the length and density of the current manuscript, we considered it more suitable to limit this investigation to a discussion paragraph against the literature, rather than adding a limited analysis to the study that would make the manuscript even longer and more difficult to follow.

**P39, L877**

*“While this study presents novel techniques to develop state-of-the-art neuromuscular models as MN-driven pools of MU Hill-type actuators, it remains to assess the performance of these more physiological and complex models compared to the more common phenomenological single-input single-actuator approaches, like the standard bEMG-driven Hill-type models (Pizzolato et al., 2015). Almost no studies that proposed models of MU pools performed this comparative assessment, which was therefore never reviewed (Raikova et al., 2023). Only two studies compared, for limited contraction tasks, the force outputs of a model of MU pool and of a single muscle-scale model, using Hill-type (Hatze, 1978) and twitch-type (Thompson et al., 2018) approaches. Both reached the same conclusions. The models of MU pool and the single-actuator muscle-scale models can yield equally accurate predictions of muscle force in the time domain, although the MU pool model always performs better in the frequency domain. Contrary to single-actuator models, models of MU pool can predict the higher frequency force fluctuations, crucial in the analysis of steadiness for example. This is explained by the superiority as control signals of vectors of MU spike trains, immune to waveform cancellation, over rectified and smoothed bEMG signals. It is worth noting that the single-actuator Hill-type model in Hatze (1978) is significantly more complex and elaborate in its phenomenological description of the MU dynamics than traditional EMG-driven Hill-type models, as reviewed (Caillet et al., 2022a), while the single-actuator twitch-type model in Thompson et al. (1978) was fully calibrated to match the force traces. It is therefore possible that MN-driven models of MU pool behave more accurately than more standard single-actuator bEMG-driven approaches, especially between contractions where the neural strategy changes, e.g., between different contraction intensities or when fatigue occurs, considering that calibrated bEMG-driven models are known to behave poorly at other activation levels (Perreault et al., 2003). In this respect, cumulative spike train (CST)-driven single-actuator models (Sartori et al., 2017), that describes the MU pool recruitment and firing strategies, were shown to more accurately predict muscle forces than classic bEMG-driven Hill-type approaches.*

*Consequently, the field requires a systematic assessment of the comparative performance of standard single-actuator neuromuscular models and models of MU pools in a variety of contraction tasks and in generic, subject-specific, and calibrated approaches. Finally, beyond the prediction accuracy, MN-driven models of MU pools provide detailed insights into the muscle’s internal dynamics and finds practical applications that standard single-actuator bEMG-driven models cannot provide, as later discussed.”*

Specific comments

P10 line 186. It is unclear whether duplicates of motor units were automatically or manually removed.

We clarified this aspect adding this sentence (P10 L187)

*“After the automatic identification of the motor units, duplicates were automatically removed according to a threshold of 30% of discharge times in common between identified spike trains (Caillet et al., 2023), and all the motor unit spike trains were visually checked for false positives and false negatives (Del Vecchio et al., 2020).”*

P13 line 260. Mus should be Mus

The correction was applied in the manuscript.

P15 line 323-324. The state variables have not been previously stated.

We addressed this comment by specifying the state variables (P16, L329)

*“With these assumptions, and after normalizing the force* $F^{M}$ *and MU length* $l$ *state variables to the* $F_{0}^{M}$ *and* $l_{0}^{M}$ *parameters and reporting them with a bar, as described in Appendix ‎A.2, the whole muscle dynamics in isometric contractions were described with EQ. 10.”*

P38 line 837. “true neural to muscle” missing a word?

We added the missing word ‘drive’ to the manuscript.

**Reviewer #2:**

The authors have developed a complex computational model, supported by experimental work, that is controlled at the level of individual motor units (MUs) and accounts for the dynamics of MU recruitment.

We thank the reviewer for their careful review of our manuscript.

The authors include some subject-specific metrics (muscle volumes, centroid lines of action) extracted from MRI. How big an effect do these subject-specific parameters have on the predicted results, as compared to generic values (i.e., how much value does this additional customization add to the model performance)?

The reviewer raises an important point. One motivation for including subject-specific metrics extracted from MRI was to develop a neuromuscular model that would be state-of-the-art on all three aspects of motor control, neuromuscular modelling, and MSK modelling by systematically using the most advanced modelling approaches available in the literature, or developing novel approaches. However, even though automated tools are available (Modenese & Renault, 2021), developing subject-specific MSK models remains time-consuming and challenging because of the necessity of MRI acquisition, MRI segmentation, and manipulations in NMSBuilder (Valente et al., 2017) to define the muscles’ lines of action.

To address the reviewer’s comment and justify this layer of modelling complexity, we added a paragraph of discussion (P44 L1023) in the subsection ‘Interfacing the fields of subject-specific motor control, neuromuscular modelling, and MSK modelling’ of the Discussion Section. According to the literature, MSK model predictions can be sensitive to the uncertainties in parameter identification and MSK architecture (Valente et al., 2014; Navacchia et al., 2016). Therefore, we now compare in this paragraph the values of key MSK parameters (optimal fibre length, tendon slack length, maximum isometric force, moment arm) extracted from the subject-specific MSK model, a generic available model of the lower limbs (Rajagopal et al., 2016), and the same model scaled to the subject’s anthropometry. We also assess how those differences in parameter values between models propagate to the predicted results. As expected from the literature, large differences were obtained between models. For example, the subject-specific TA maximum isometric force was 15% lower than the available generic values (Rajagopal et al., 2016), which would have linearly affected the amplitude of the predicted whole muscle force by 15%, as now discussed in the manuscript.

**P44 L1023**

*“MSK model predictions can be sensitive to the uncertainties in parameter identification and MSK architecture (Valente et al., 2014; Navacchia et al., 2016). The subject-specific properties of the TA muscle used in this study were compared to those from a generic published model (Rajagopal et al., 2016), and the same model scaled to the anthropometry of the subject with the Opensim built-in tools (Delp et al., 2007; Seth et al., 2018). The values of the optimal length* $l_{0}^{M}$ *and tendon slack length* $l_{s}^{T}$ *did not vary between the three models (<3% variation). The subject-specific muscle-tendon length* $l^{MT}$ *at 30° plantarflexion was 3% shorter and 12% longer than in the scaled and generic models, respectively. Considering the simplification of rigid tendon, these differences in length linearly propagate to the estimation of the normalized MU length* $\bar{l}$ *and nonlinearly to the FL and length-dependent activation dynamics of the MUs (Figure 4). The subject-specific MRI-based maximum isometric force* $F_{0}^{M}$ *was 15% lower than proposed in the generic model. This difference linearly propagates to the linear scaling of the distribution of MU isometric forces* $f_{0}^{MU}$ *in EQ. 8 (Figure 7B-D) and to the amplitude in N of the predicted whole muscle force (Figure 8). The highest difference was obtained for the muscle moment arm with the ankle joint in dorsiflexion, where the subject-specific quantity was 33% and 40% lower than the scaled and generic ones. This difference linearly propagates to the estimation of experimental muscle force from experimental joint torque (Figure 5) that was used for model validation (Figure 8).”*

Parameters of the model are derived from a combination of calibration of some parameters, and direct measurement and application of others. As such, a true model validation has not been performed. Please include discussion of this limitation in the manuscript.

The model does not integrate any parameter that was calibrated by minimizing some external cost function that would involve the recorded experimental data (HDEMG signals or joint torque). In this way, the proposed model diverges from the classic EMG-driven approach where key neuromuscular parameters (usually optimal fibre length, tendon slack length, maximum isometric force, activation dynamics parameters) are calibrated by minimizing the difference between predicted and independently measured joint torques.

In our model, all parameters are either:

- Directly derived from the experimental literature (distribution of MU maximum isometric forces, parameters in the ODEs defining the excitation and activation dynamics…). We gave great attention, when possible, to only consider human or mammalian literature data (P39 L859-876, for example) to define the parameters included in our relationships, with the aim to maintain internal consistency in our model.
- Directly measured from the subject-specific MSK model that was obtained from MRI data.

The great attention taken to derive those parameters aimed to avoid the need for parameter calibration. Consequently, all parameters were derived independently from the HDEMG and Torque data that we used to control and validate the model.

The only parameter we calibrated was the MN size, necessary for the reconstruction of the MU pool (third test case in Figures 6 to 8 in the manuscript). In this case, the MN size calibration is used to derive the continuous distribution of MN electrophysiological properties across the MU pool, with the aim of data augmentation. The reconstructed vector of MN spike trains is then input to the non-calibrated neuromuscular model. This identification of the MN size parameter was required in only one of the three types of predictions proposed in our manuscript (red traces in Figure 8 only).

Consequently, as the model only integrated parameters obtained from the experimental literature and direct subject-specific measurements, we believe that a true model validation was performed, like in the majority of the published Hill-type models. To address this comment, we clarified the manuscript by rewording and extending the following discussion:

**P44 L1040:**

*“The model proposed in this study and the predictions displayed in Figure 8 did not rely on any parameter calibration that would minimize external cost functions, like commonly proposed in EMG-driven approaches where some subject-muscle-specific properties are, for example, calibrated by minimizing the difference between predicted and experimental joint torques (Lloyd & Besier, 2003; Pizzolato et al., 2015).*

*Beyond the MSK parameters directly measured with the subject-specific MSK model, it is worth noting that the remaining parameters in the equations that define the model’s force-generating properties (EQ. 8 and EQ. 11 to EQ. 18) are fitting experimental data from the literature, that are independent from the EMG and torque signals that were used in this study to control and validate the model.*

*With this subject-specific and MN-driven approach, the accurate prediction of the whole muscle force amplitude at 30% MVC in Figure 8E-G were consequently obtained without parameter calibration owing to a physiological and comprehensive description of the neural drive (Figure 6), which the bEMG envelopes normalized by MVC signals cannot achieve, an adequate distribution of the MU’s maximum isometric forces* $f_{0,k}^{MU}$ *across the modelled MU population (Figure 7), and an estimation of muscle co-contraction during ankle dorsiflexion (Figure 5), as discussed.*

*In cases for which subject-specific measurements are not possible or muscle-specific data are not as available in the literature as for the TA, which is the case for most human muscles (Duchateau & Enoka, 2022), missing muscle architectural parameters or the coefficients in EQ. 8 could be included in an optimization routine aiming to minimize the difference between experimental and predicted joint torques, for example.*

*The only calibrated parameter in the pipeline in Figure 1 was the size of the experimentally identified MNs, that was required for the specific test case of complete reconstruction of the MN pool (Figure 6 and Figure 8, red traces) to derive the continuous distribution of electrophysiological properties across the population of MN LIF models. Again, this parameter identification was independent from the experimental measurements of joint torque used in this study to validate the model. The two other approaches taken to predict the muscle force solely relied on the experimental samples of identified MNs (Figure 8, blue and traces) and did not require the calibration of the MN size parameter.”*

Lines 621-622: What was the reason for these inaccurate description of discharge activity?

The dataset the reviewer refers to, that was obtained in another published study (Caillet et al., 2023), only included 3 identified discharging MUs, because few electrodes (12 electrodes, 12mm interelectrode distance) were used to record the EMG signals at 50% MVC. In that study, it is explained that the 3 identified MUs are high-threshold MUs. Consequently, the discharging activity of the MU pool was not decoded for most of the range of force generation. As discussed in the manuscript, the experimental datasets of identified MUs must be sufficiently representative of the discharge activity of the MU pool (sometimes assessed with the coherence metric (Caillet et al., 2022b)) to provide accurate predictions of muscle force. The manuscript was adapted to clarify this point.

**P28 L625**

*“The dataset of three identified MUs was disregarded in the following results because too few and only high-threshold MUs were identified in this dataset, which therefore provided a highly inaccurate description of the full spectrum of MU properties and discharge activity of the real MU pool (Caillet et al., 2022b).”*

Why were experimental samples better approximated at lower MVCs (30% versus 50%)?

The reviewer raises an important question that should be clarified in the manuscript to improve the reader’s understanding of the sensitivity of the proposed model to the quality of the input experimental data. In the study where the experimental motoneuronal data was derived (Caillet et al., 2023), we explain that the current source-separation algorithms are biased towards identifying high-threshold ‘large’ MUs that contribute to most of the EMG signal. At higher contraction intensities (50% MVC), those large MUs are recruited and ‘mask’ the smaller MUs that develop less energy. Although better results (higher numbers of ‘small’ MUs) can be obtained at 30% MVC by increasing the electrode density and sampling the action potentials over larger number of electrodes, this approach does not improve the results at 50% MVC (Caillet et al., 2023). These are the reasons why the experimental samples were better approximated at lower MVCs.

This was clarified in the manuscript:

**P28 L633** (Results) and **P42 L965** (Discussion)

*“Higher heterogeneity and lower representation of low-threshold MUs were obtained with shallower grids and at 50% MVC. Classically, the decomposition algorithms converge towards the large MUs that contribute most to the EMG signals, while action potentials of the small MUs of lower energy are masked by the potential of larger units, more of which are recruited at 50% MVC than at 30% MVC. At 30% MVC, increasing the electrode density better samples the action potential profiles of the low-threshold unmasked MUs across multiple electrodes, enabling their identification.”*

It would be informative if the authors discussed applications where they expect their model to perform better than current models, and why (e.g., phenomenological models). The authors do not provide any benchmarking against more simplistic models, therefore it is difficult to assess if the additional complexity is warranted, or if the additional effort and complexity required has a negligible outcome on model performance. Please include some numerical values to describe the computational costs associated with the model (i.e., simulation time for a specific set of hardware) as compared to the classic single-input Hill-type actuators.

We understand the three concerns raised by the reviewer (1. Insufficient discussion of the possible applications where the MU pool model is expected to be more suitable than standard single-actuator models, 2. No benchmarking against more simplistic models in the assessment of model performance, and 3. No analysis of the relative computational cost.). We addressed these comments in the new version of the manuscript by comparing the model complexity and computational cost to those of simpler models, and justified the added complexity with regards to model performance and possible applications. (The relevant sections of the revised manuscript are sequentially reproduced below, after our response to the reviewer).

1. **Modelling complexity justified by future perspectives and specific applications (P45, L1068)**

The reasons for the complexity of our model were not sufficiently and clearly justified in light of possible specific applications. We addressed this comment in the new version of the manuscript by gathering all previous scattered occurrences of such discussion in a new dedicated subsection of the Discussion section ‘Applications and futures perspectives’ (P45, L1068). In this new section, the discussion is now enriched with examples of specific applications where of MN-driven of MU pool would be more adequate than a standard single-actuator bEMG-driven model. Specific examples were given in the fields of human-machine interfacing, biomechanics, neurophysiology, muscle physiology, and muscle neuromechanics.

2. **Benchmarking against simplistic models (P39, L877)**

We agree with the reviewer that benchmarking the prediction accuracy of our model against classic single-actuator bEMG-driven models is important. This is the reason why we have been preparing a separate study in which we are undertaking a systematic comparative assessment of MU pool and simpler single-actuator models. To address the reviewer’s comment, we added a new paragraph (P39, L877) to the section where we discuss how our model fits into the context of existing models. In this paragraph, we now review the literature that provides comparative assessments of MU pool models against single-actuator muscle-scale models. We come to the conclusion that MU pool models are expected to provide more accurate predictions of muscle force in the frequency domain, while the literature indicates that similar performance can be expected in the time domain. However, other evidence suggest that MU pool models might be more accurate in the time domain in specific contraction tasks where different neural strategies are involved.

It is also recalled in this new paragraph that the proposed MU pool model is not limited to predictions of muscle force from individual motoneuronal activity. It also provides a window into the muscle’s internal dynamics that cannot be observed *in vivo*, and finds dedicated applications as addressed in the reviewer’s previous comment.

As now discussed in this new paragraph, the literature that performs comparative analysis of MU pool and single-actuator models is very scarce. Therefore, the field requires a systematic comparative study that involves a variety of contraction tasks, types of inputs, and variants of Hill-type models to derive robust conclusions. Considering the length and density of the current manuscript, we considered it more suitable to limit this investigation to a discussion paragraph against the literature, rather than adding a limited analysis to the study that would make the manuscript even longer and more difficult to follow.

3. **Analysis of the computational cost (P47, L1133)**

As suggested by the reviewer, we now indicate in the manuscript numerical values to describe the computational costs associated with the model (i.e., simulation time for the specific set of hardware we used) as compared to the classic single-input Hill-type actuators. We additionally provide solutions to drastically reduce the computational load without degrading the prediction accuracy of the muscle force.

**P45, L1068:**

*“Applications and future perspectives*

*The MN-driven multi-MU model proposed in this study comprehensively describes the recruitment and firing strategy of the MU pool during human voluntary muscle contractions. For these reasons, this detailed model finds applications in answering scientific questions where the lump dynamics of the classic single-actuator bEMG-driven Hill-type models provide limited help.*

*In the study of the human muscle architecture, by combining the aforementioned mapping of the MU pool in volumetric muscle representations with recently developed ultrasound measurement techniques for tracking MU twitches and mechanical properties (Lubel et al., 2022; Lubel et al., 2023), one could gain insights into the distribution of MU territories within human muscles, which remains an open question. The volumetric mapping of MUs also provides a convenient framework for modelling the transversal mechanical interactions between MUs (Herzog, 2019), and the resulting force-varying load path within the muscle tissue, that results in a nonlinear summation of the individual MU forces.*

*In the field of neurophysiology, the MN-driven model of MU population proposed in this study is suited for integrating MN synergies in simulations. Contrary to single-input Hill-type models that lump the dynamics of synaptic input, MN recruitment, and MN discharge into a single phenomenological signal, the MU pool modelled in Figure 1 can be divided into functional clusters and receive different common inputs to reduce the dimensionality of the control (Hug et al., 2023). For example, in multi-muscle MSK models with MU-actuated volumetric representations of muscles, the model proposed in Figure 1 is also suited for the investigation of neural synergies between muscles, where MN clusters span across muscles (Hug et al., 2023), with strategies specific to the muscle groups (Del Vecchio et al., 2023).*

*In the study of human muscle contraction dynamics, the model developed in this study provides a credible window onto the distribution of the excitation-contraction dynamics of individual MUs across the MU pool (Figure 4) that cannot be measured in human in vivo, and advances our understanding of the muscle-specific neuromechanical strategies for muscle force generation. For example, Figure 4 suggests that, at 30% MVC, the highest-threshold recruited MUs with the largest force-generating capacities are not those producing the highest forces within the recruited pool due to low activation states explained by the onion skin theory (Figure 8A, D). As the model integrates realistic descriptions of the MU-specific firing, recruitment, excitation, and activation dynamics responsible for muscle force generation, it also provides a convenient framework for integrating and investigating MU-related mechanisms, such as the effect of fatigue on the MU firing and recruitment strategies (Taylor et al., 2016) or other fatigue-induced changes in MU activation dynamics as already proposed in single-MU models (Callahan 2016, Shorten 2007).*

*However, some currently investigated limitations can prevent the proposed MN-driven MU-based model from being readily applicable to the aforementioned scientific questions. First, although the pipeline in Figure 1 is fully suited for dynamic contractions, the study proposed here was constrained to isometric tasks due to current experimental limitations in the decomposition of HDEMG signals into MU spike trains during motion. New computational approaches are emerging to identify individual spike trains in quasi-static (Oliveira & Negro, 2021) and dynamic (Glaser & Holobar, 2018) tasks, where 7-20 MUs can be currently identified. Second, data acquisition remains challenging and time-consuming. As opposed to straightforward bEMG recording and filtering, the pipeline in Figure 1 requires decomposing HDEMG signals and manually editing the identified spike trains editing, while taking precautions to identify the full spectrum of discharging motor units for accurate predictions (Figure 6), as discussed. These challenges should be addressed with the rapid emergence of guidelines for manufacturers on HDEMG grid design (Caillet et al., 2023), open-source tools for automatic HDEMG decomposition and spike trains edition (Avrillon et al., 2023), automated spike train identification approaches based on machine learning (Clarke et al., 2020) and blind-source separation methods (Rossato et al., 2023), and MU pool reconstruction methods (Caillet et al., 2022b).”*

**P39, L877**

*“While this study presents novel techniques to develop state-of-the-art neuromuscular models as MN-driven pools of MU Hill-type actuators, it remains to assess the performance of these more physiological and complex models compared to the more common phenomenological single-input single-actuator approaches, like the standard bEMG-driven Hill-type models (Pizzolato et al., 2015). Almost no studies that proposed models of MU pools performed this comparative assessment, which was therefore never reviewed (Raikova et al., 2023). Only two studies compared, for limited contraction tasks, the force outputs of a model of MU pool and of a single muscle-scale model, using Hill-type (Hatze, 1978) and twitch-type (Thompson et al., 2018) approaches. Both reached the same conclusions. The models of MU pool and the single-actuator muscle-scale models can yield equally accurate predictions of muscle force in the time domain, although the MU pool model always performs better in the frequency domain. Contrary to single-actuator models, models of MU pool can predict the higher frequency force fluctuations, crucial in the analysis of steadiness for example. This is explained by the superiority as control signals of vectors of MU spike trains, immune to waveform cancellation, over rectified and smoothed bEMG signals. It is worth noting that the single-actuator Hill-type model in Hatze (1978) is significantly more complex and elaborate in its phenomenological description of the MU dynamics than traditional EMG-driven Hill-type models, as reviewed (Caillet et al., 2022a), while the single-actuator twitch-type model in Thompson et al. (1978) was fully calibrated to match the force traces. It is therefore possible that MN-driven models of MU pool behave more accurately than more standard single-actuator bEMG-driven approaches, especially between contractions where the neural strategy changes, e.g., between different contraction intensities or when fatigue occurs, considering that calibrated bEMG-driven models are known to behave poorly at other activation levels (Perreault et al., 2003). In this respect, cumulative spike train (CST)-driven single-actuator models (Sartori et al., 2017), that describes the MU pool recruitment and firing strategies, were shown to more accurately predict muscle forces than classic bEMG-driven Hill-type approaches.*

*Consequently, the field requires a systematic assessment of the comparative performance of standard single-actuator neuromuscular models and models of MU pools in a variety of contraction tasks and in generic, subject-specific, and calibrated approaches. Finally, beyond the prediction accuracy, MN-driven models of MU pools provide detailed insights into the muscle’s internal dynamics and finds practical applications that standard single-actuator bEMG-driven models cannot provide, as later discussed.”*

**P47, L1133**

*“Finally, with four ODEs to solve at each time step for each of the modelled motor units, the model proposed in this study is currently computationally too expensive for real-time applications, as opposed to the classic single-input Hill-type actuators, which usually include zero to two ODEs. For the simulation of a 40s-long muscle contraction using a standard laptop (RAM: 12 GB, CPU: one Intel Core i7-1165G7 2.80 GHz), the pipeline in Figure 1 implemented in Python ran in 18 minutes for a MU pool of* $N_{r}=16$ *MUs (Figure 8A, dotted trace), in 63 minutes for* $N_{r}=81$ *MUs (Figure 8C, dotted trace), and in 240 to 300 minutes for the complete pool of* $N=400$ *MUs (Figure 8, red traces), that additionally required preliminary MU pool reconstruction. For comparison, a single-actuator rigid-tendon Hill-type model (Millard et al., 2013) ran in less than a minute on the same machine for the same 40s-long trapezoidal isometric contraction where the HDEMG signals were averaged and normalized to MCV signals. Besides implementations of the model in a faster compiled programming language (for example C++) and using better performing machines, the computational speed could be drastically increased by using parallel computing to solve the independent MU dynamics, by considering smaller reconstructed populations of 50 MUs without loss of accuracy in the estimated neural drive to muscle, as previously discussed (Caillet et al., 2022b), or by, for example, simplifying the current model of MU excitation and activation dynamics with analytical descriptions of the fibre APs and calcium transients instead of the two 2nd-order ODEs in EQ. 13 and EQ. 14.”*

REFERENCE LIST

Avrillon S, Hug F, Gibbs C, Farina D (2023) Tutorial on MUedit: An open-source software for identifying and analysing the discharge timing of motor units from electromyographic signals. bioRxiv 2023.07. 13.548568.

Caillet AH, Phillips AT, Carty C, Farina D, Modenese L (2022a) Hill-type computational models of muscle-tendon actuators: A systematic review. bioRxiv, doi: https://doi.org/10.1101/2022.10.14.512218

Caillet AH, Phillips AT, Farina D, Modenese L (2022b) Estimation of the firing behaviour of a complete motoneuron pool by combining electromyography signal decomposition and realistic motoneuron modelling. PLOS Computational Biology 18:e1010556.

Caillet AH, Avrillon S, Kundu A, Yu T, Andrew T.M. Phillips, Modenese L, Farina D (2023) Larger and denser: An optimal design for surface grids of EMG electrodes to identify greater and more representative samples of motor units. eNeuro ENEURO.0064-23.2023.

Clarke AK, Atashzar SF, Del Vecchio A, Barsakcioglu D, Muceli S, Bentley P, Urh F, Holobar A, Farina D (2020) Deep learning for robust decomposition of high-density surface EMG signals. IEEE Transactions on Biomedical Engineering 68:526-534.

Del Vecchio A, Germer CM, Kinfe TM, Nuccio S, Hug F, Eskofier B, Farina D, Enoka RM (2023) The forces generated by agonist muscles during isometric contractions arise from motor unit synergies. Journal of Neuroscience .

Delp SL, Anderson FC, Arnold AS, Loan P, Habib A, John CT, Guendelman E, Thelen DG (2007) OpenSim: Open-source software to create and analyze dynamic simulations of movement. TBME 54:1940-1950.

Duchateau J, Enoka RM (2022) Distribution of motor unit properties across human muscles. J.Appl.Physiol. 132:1-13.

Farina D, Vujaklija I, Brånemark R, Bull AMJ, Dietl H, Graimann B, Hargrove LJ, Hoffmann K, Huang H, Ingvarsson T, Janusson HB, Kristjánsson K, Kuiken T, Micera S, Stieglitz T, Sturma A, Tyler D, Weir RFf, Aszmann OC (2021) Toward higher-performance bionic limbs for wider clinical use. Nature biomedical engineering 1-13.

Farina D, Vujaklija I, Sartori M, Kapelner T, Negro F, Jiang N, Bergmeister K, Andalib A, Principe J, Aszmann OC (2017) Man/machine interface based on the discharge timings of spinal motor neurons after targeted muscle reinnervation. Nature biomedical engineering 1:1-12.

Glaser V, Holobar A (2018) Motor unit identification from high-density surface electromyograms in repeated dynamic muscle contractions. IEEE Transactions on Neural Systems and Rehabilitation Engineering 27:66-75.

Hatze H (1980) Myocybernetic Control Models of Skeletal Muscle - Characteristics and Applications. University of South Africa Press, Pretoria, .

Hatze H (1978) A general myocybernetic control model of skeletal muscle. Biological Cybernetics 28 143-157.

Herzog W (2019) The problem with skeletal muscle series elasticity. BMC biomedical engineering 1:28.

Hug F, Avrillon S, Ibáñez J, Farina D (2023) Common synaptic input, synergies and size principle: Control of spinal motor neurons for movement generation. J.Physiol.(Lond.) 601:11-20.

Kaufman KR, An K, Litchy WJ, Chao E (1991) Physiological prediction of muscle forces—I. theoretical formulation. Neuroscience 40:781-792.

Lloyd DG, Besier TF (2003) An EMG-driven musculoskeletal model to estimate muscle forces and knee joint moments in vivo. Journal of Biomechanics 36:765-776.

Lubel E, Grandi Sgambato B, Rohlen R, Ibanez J, Barsakcioglu D, Tang M, Farina D (2023) Non-linearity in motor unit velocity twitch dynamics: Implications for ultrafast ultrasound source separation. bioRxiv 2023.03. 24.533983.

Lubel E, Sgambato BG, Barsakcioglu DY, Ibáñez J, Tang M, Farina D (2022) Kinematics of individual muscle units in natural contractions measured in vivo using ultrafast ultrasound. Journal of Neural Engineering 19:056005.

Millard M, Uchida T, Seth A, Delp SL (2013) Flexing computational muscle: Modeling and simulation of musculotendon dynamics. Journal of biomechanical engineering 135:021005.

Modenese L, Renault J (2021) Automatic generation of personalised skeletal models of the lower limb from three-dimensional bone geometries. J.Biomech. 116:110186.

Modenese L, Kohout J (2020) Automated generation of three-dimensional complex muscle geometries for use in personalised musculoskeletal models. Ann.Biomed.Eng. 48:1793-1804.

Navacchia A, Myers CA, Rullkoetter PJ, Shelburne KB (2016) Prediction of in vivo knee joint loads using a global probabilistic analysis. J.Biomech.Eng. 138:031002.

Oliveira AS, Negro F (2021) Neural control of matched motor units during muscle shortening and lengthening at increasing velocities. J.Appl.Physiol. 130:1798-1813.

Perreault EJ, Heckman CJ, Sandercock TG (2003) Hill muscle model errors during movement are greatest within the physiologically relevant range of motor unit firing rates. J.Biomech. 36:211-218.

Pizzolato C, Lloyd DG, Sartori M, Ceseracciu E, Besier TF, Fregly BJ, Reggiani M (2015) CEINMS: A toolbox to investigate the influence of different neural control solutions on the prediction of muscle excitation and joint moments during dynamic motor tasks. J.Biomech. 48:3929-3936.

Raikova R, Krutki P, Celichowski J (2023) Skeletal muscle models composed of motor units: A review. Journal of Electromyography and Kinesiology 102774.

Rajagopal A, Dembia CL, DeMers MS, Delp DD, Hicks JL, Delp SL (2016) Full-body musculoskeletal model for muscle-driven simulation of human gait. TBME 63:2068-2079.

Rossato J, Hug F, Tucker K, Lacourpaille L, Farina D, Avrillon S (2023) I-spin live: An open-source software based on blind-source separation for decoding the activity of spinal alpha motor neurons in real-time. bioRxiv 2023.04. 14.536933.

Sartori M, Yavuz U, Farina D (2017) In vivo neuromechanics: Decoding causal motor neuron behavior with resulting musculoskeletal function. Scientific reports 7:13465-14.

Seth A, Hicks JL, Uchida TK, Habib A, Dembia CL, Dunne JJ, Ong CF, Demers MS, Rajagopal A, Millard M, Hamner SR, Arnold EM (2018) OpenSim: Simulating musculoskeletal dynamics and neuromuscular control to study human and animal movement. PLoS computational biology 14:e1006223.

Taylor JL, Amann M, Duchateau J, Meeusen R, Rice CL (2016) Neural contributions to muscle fatigue: From the brain to the muscle and back again. Med.Sci.Sports Exerc. 48:2294.

Thompson CK, Negro F, Johnson MD, Holmes MR, McPherson LM, Powers RK, Farina D, Heckman CJ (2018) Robust and accurate decoding of motoneuron behaviour and prediction of the resulting force output. J.Physiol.(Lond.) 596:2643-2659.

Valente G, Crimi G, Vanella N, Schileo E, Taddei F (2017) nmsBuilder: Freeware to create subject-specific musculoskeletal models for OpenSim. Comput.Methods Programs Biomed. 152:85-92.

Valente, G.,Pitto, L.,Testi, D.,Seth, A.,Delp, S.L.,Stagni, R.,Viceconti, M.,Taddei, F.(2014) Are subject-specific musculoskeletal models robust to the uncertainties in parameter identification? PloS one, 9, e112625. https://www.ncbi.nlm.nih.gov/pubmed/25390896.

Woittiez RD, Huijing PA, Boom H, Rozendal RH (1984) A three‐dimensional muscle model: A quantified relation between form and function of skeletal muscles. J.Morphol. 182:95-113.
